# Clinical experiences of delayed contrast enhancement with cardiac computed tomography: case series

**DOI:** 10.1186/1756-0500-6-2

**Published:** 2013-01-03

**Authors:** Manavjot S Sidhu, Brian B Ghoshhajra, Shanmugam Uthamalingam, Niamh Kilcullen, Leif-Christopher Engel, Hector M Medina, Vikram Venkatesh, Yongkasem Vorasettakarnkij, Udo Hoffmann, Ricardo C Cury, Suhny Abbara, Thomas J Brady

**Affiliations:** 1 Department of Cardiovascular Imaging, Cardiac MR PET CT Program, Department of Radiology and Division of Cardiology, Massachusetts General Hospital and Harvard Medical School, 165 Cambridge Street, Suite 400, Boston, MA, 02114, USA

## Abstract

**Background:**

Myocardial delayed enhancement (MDE) by gadolinium-enhanced cardiac MRI is well established for myocardial scar assessment in ischemic and non-ischemic heart disease. The role of MDE by cardiac CT (CT-MDE) is not yet defined.

**Findings:**

We reviewed all clinical cases of CT-MDE at a tertiary referral center to present the cases as a case series. All clinical cardiac CT exams which utilized CT-MDE imaging between January 1, 2005 and October 1, 2010 were collected as a series and their findings were also compared with available myocardial imaging to assess for myocardial abnormalities, including echocardiography (wall motion, morphology), cardiac MRI (delayed enhancement, morphology), SPECT MPI (perfusion defects). 5,860 clinical cardiac CT exams were performed during the study period. CT-MDE was obtained in 18 patients and was reported to be present in 9 patients. The indications for CT-MDE included ischemic and non-ischemic heart diseases. In segments positive for CT-MDE, there was excellent agreement of CT with other modalities: echocardiography (n=8) demonstrated abnormal morphology and wall motion (k=1.0 and k=0.82 respectively); prior MRI (n=2) demonstrated abnormal delayed enhancement (MR-MDE) (k=1.0); SPECT MPI (n=1) demonstrated fixed perfusion defects (k=1.0). In the subset of patients without CT-MDE, no abnormal segments were identified by echocardiography (n=8), MRI (n=1) and nuclear MPI (n=0).

**Conclusions:**

CT-MDE was performed in rare clinical situations. The indications included both ischemic and non-ischemic heart disease and there was an excellent agreement between CT-MDE and abnormal myocardium by echocardiography, cardiac MRI, and nuclear MPI.

## Findings

We reviewed all clinical cases of CT-MDE at a tertiary referral center to present the cases as a case series. All clinical cardiac CT exams which utilized CT-MDE imaging between January 1, 2005 and October 1, 2010 were collected as a series and their findings were also compared with available myocardial imaging to assess for myocardial abnormalities, including echocardiography (wall motion, morphology), cardiac MRI (delayed enhancement, morphology), SPECT MPI (perfusion defects) 5,860 clinical cardiac CT exams were performed during the study period. CT-MDE was obtained in 18 patients and was reported to be present in 9 patients. The indications for CT-MDE included ischemic and non-ischemic heart diseases. In segments positive for CT-MDE, there was excellent agreement of CT with other modalities: echocardiography (n=8) demonstrated abnormal morphology and wall motion (k=1.0 and k=0.82 respectively); prior MRI (n=2) demonstrated abnormal delayed enhancement (MR-MDE) (k=1.0); SPECT MPI (n=1) demonstrated fixed perfusion defects (k=1.0). In the subset of patients without CT-MDE, no abnormal segments were identified by echocardiography (n=8), MRI (n=1) and nuclear MPI (n=0).

### Introduction

Cardiac magnetic resonance imaging (MRI) with gadolinium can detect myocardial delayed enhancement (MDE) [1] and has become the clinical imaging standard for the evaluation of myocardial infarction and scar. MDE is also useful in identifying myocardial injury and in the assessment of infiltrative, inflammatory diseases of the heart , ischemic heart disease and even in cardiac neoplasm [2]. However, there are numerous contraindications to cardiac MRI, including severe claustrophobia, several arrhythmias, metal implants and severe renal insufficiency (a risk factor for nephrogenic systemic fibrosis). In these patients, the clinical need for an alternative diagnostic tool for scar/injury imaging occasionally arises.

Cardiac computed tomography (CT) can identify MDE with iodinated contrast material because the pharmacokinetics of iodinated agents are very similar to those of gadolinium-DTPA [3,4]. Both agents are retained in tissue with an increased volume of distribution including myocardial infarction and scar. A number of studies are underway to assess the role of cardiac CT using MDE. In this report, we review our clinical experience where CT-MDE was performed to yield insights into application of this novel technique.

We retrospectively reviewed all clinical cases of CT-MDE at a tertiary care referral center to present our experience as a series of cases.

## Methods

### Study design

A waiver was obtained from our Institutional Review Board (Partners Healthcare IRB) for this retrospective study. All clinical cardiac CT exams performed from January 1, 2005 to October 1, 2010 were included, regardless of indication. Cases performed for research purposes were excluded.

The text of all reports was screened for the use of a delayed enhancement protocol. Reports were then manually reviewed to refine the list to those patients who underwent delayed imaging purely for the purpose of MDE assessment. For example, early or immediate delayed phase images obtained for thrombus detection in the cardiac chambers were excluded (i.e. 2-minute delayed scans for atrial appendage thrombus exclusion). The cardiac CT images, reports and electronic records were also reviewed for technique, technical parameters (tube voltage, tube current, gating, timing, slice thickness), radiation dose (dose length product as obtained from the dose exposure record), and image quality (subjective assessment as dictated by interpreting reader). Images were analyzed to determine the contrast-to-noise ratio using regions of interest in the abnormal myocardium and the remote normal myocardium.

Using the AHA 17 segment model [5], myocardial segments on CT images were then identified. To corroborate CT-MDE findings in patients, all cardiac imaging modalities performed within a period of three months were retrieved and reviewed. These included 2-dimensional echocardiography, cardiac MRI, and single photon emission cardiac tomogram (SPECT) myocardial perfusion imaging (MPI) scans. Clinical reports were reconciled into standardized segmental readings by physicians with appropriate training in cardiac CT and MRI (B.B.G & S.U.), 2-dimensional echocardiography (N.K.), and nuclear imaging (S.U.).

### Imaging methods

Cardiac CT was performed with 64-detector-row and higher multidetector single- and dual-source scanners (Somatom Sensation 64, Somatom Definition, and Somatom Definition Flash; Siemens, Forchheim, Germany). Standard parameters were set for arterial phase scans for image acquisition as per clinical protocol. All scans were performed using the timing bolus method to determine contrast material transit time, usually with a 20 mL standard test bolus dose of the contrast medium at 4-7 mL/second, followed by 20-40 mL of normal saline flush. An intravenous bolus of contrast agent, tailored to scan parameters, usually 60-90 mL (iopamidol 76% [370 mg of iodine per milliliter] Isovue; Bracco Diagnostics, Princeton, New Jersey was administered by using a dual-head power injector and was usually followed by 40 mL of normal saline at 4-7 mL / sec. Tube voltage and tube current were varied according to the patient size and institutional protocols.

Additional contrast was given after the arterial phase scan at a slow rate of up to 2 mL / second, usually between 50 and 90 mL, for a total of approximately 150 mL. The amount, rate, and timing were determined on a case-by-case basis by the supervising physician.

MDE acquisitions were performed after a variable period of 5-10 minutes after the initial contrast administrations (mean delay time 7.0 minutes), at physician discretion. To minimize the radiation exposure and increase the myocardial contrast, decreased tube voltage (100 kVp or 80 kVp) and prospective ECG triggering were used for the delayed acquisition in all patients.

Routine clinical 2-dimensional echocardiograms, SPECT-MPI images, and cardiac MRI exams were acquired according to standard institutional protocols.

### Data analysis

Axial source CT images were transferred to a dedicated image processing workstation (Osirix MD v 1.0.1, Pixmeo, Bernex, Switzerland) for analysis. Cardiac CT clinical reports and DICOM image headers were also parsed for the technique, radiation dose, and indications. Images were analyzed on the workstation for image quality, and contrast to noise ratio in the areas of delayed enhancement. The contrast-to-noise ratio of the cardiac CT images was calculated as the signal intensity difference between enhanced and remote myocardium divided by the standard deviation of the signal intensity (attenuation) within the remote normal myocardium [6]. Total effective dose was calculated as a product of the dose-length product and a standard conversion coefficient for the chest (k=0.014 mSv/ [mGy*cm]) [7].

In cases where MDE was reported, myocardial segments on CT images were identified using the AHA 17-segment model. The segments with increased signal intensity on the delayed scan were identified as segments with MDE. Wall motion of each segment was analyzed and was visually graded as normal (0), hypokinetic (1), or akinetic/dyskinetic (2) respectively by physicians with appropriate training in cardiac CT (S.U. /B.B.G).

2-Dimensional echocardiograms (done within 3 months of the CT scans) were then retrieved and analyzed at a dedicated DICOM viewing workstation (Xcelera Philips, Michigan). Using the standardized AHA 17 segment model, the wall motion of each segment was analyzed and was visually graded as normal (0), hypokinetic (1), or akinetic/dyskinetic (2) respectively by a physician with American College of Cardiology level III training in echocardiography (N.K.). The morphology of the segment (abnormal wall thickness/abnormal echogenicity) was also analyzed and recorded on a segmental basis.

Any previous or intercurrent cardiac MRI scans (performed for any reason) were also retrieved to the image processing workstation for analysis. The segments were analyzed by physicians with appropriate training in cardiac MRI (S.U., B.B.G.) for presence or absence of MR-MDE.

SPECT-MPI images were retrieved to a dedicated viewing workstation (Invia medical imaging solutions, Corridor 4D, Michigan) and the images were analyzed for presence or absence of fixed perfusion defects. Readers of the various exams were blinded to the results of the exams.

### Statistical analysis

All continuous variables are presented as means +/- standard deviations. Agreement between CT (abnormal myocardial delayed enhancement, and separately abnormal segmental wall motion abnormality) and echocardiogram (morphology and separately LV regional wall motion) was calculated and mean kappa values were determined (<0.20 = poor agreement; 0.21–0.40= fair; 0.41 -0.60 = good; 0.61-0.80 = very good; 0.81-1.0 = excellent agreement). Agreement between CT and MRI with regard to presence of MDE was also calculated and mean kappa values were determined using the same scale. Agreement between CT and SPECT-MPI with regards to presence of fixed perfusion defects was also calculated and mean kappa values were determined using the same scale. Statistical analysis was performed using SPSS version 17 (SPSS Inc., Chicago, IL).

## Results

Of the 5,860 total cardiac clinical CT exams performed during the study period, an MDE protocol was performed in 18 cases. The characteristics of the patient population are contained in Table 1. The clinical indications for MDE protocol included assessment for the presence or progression of myocardial scarring due to ischemic heart disease (n=9), non-ischemic causes i.e. infiltrative disease (n=3), non ischemic dilated cardiomyopathy (n=3), hypertrophic cardiomyopathy (n=2), and prior surgery (n=1).

**Table 1:** Demographics and baseline characteristics (+CT MDE = positive delayed enhancement on CT, -CT MDE = no delayed enhancement on CT)

	**All**	**Positive CT MDE**	**Negative CT MDE**
Age (Years)	57 ± 17	61 ± 16	54 ± 17
Diabetes	2	1	1
Hypertension	11	6	5
Dyslipidemia	5	3	2
Medications			
Statins	8	5	3
Aspirin	13	7	6
Beta Blockers	9	5	4
CAD	8	4	4
Smoking	4	3	1
Obesity	4	2	2
Revascularization	3	1	2

A summary of the findings in each patient, in the segments with abnormal delayed enhancement on cardiac CT as well as all other available modalities is listed in Table 2.

**Table 2:** Correlation between the myocardial segments showing delayed enhancement on cardiac CT and at least one other imaging modality i.e. TTE, SPECT, or cardiac MRI

	**Clinical indication**	**Contraindication to a Cardiac MRI at the time of Cardiac CT**	**Myocardial segments with Delayed enhancement**	**Comparative imaging modality**	**Myocardial segments on comparative modality**
1	CAD -- Chest pain and inconclusive cardiac MRI. (MRI was not able to predict the cause of DE)	Anxiety causing incomplete and inconclusive Cardiac exam	Inferior wall (Transmural)	SPECT MRI	SPECT - Fixed perfusion defect in the inferior wall MRI – Delayed enhancement in Inferior wall. CTA – Obstructive disease in the dominant RCA
2	CM -- Progression of Sarcoidosis (new pacemaker precluded followup MRI)	Permanent Pacemaker	Lateral wall Apical LV wall (Patchy foci)	MRI	Lateral Apical left ventricular wall (Patchy foci)
3	CAD -- Assessment of ischemic scar	Permanent Pacemaker	Basal wall Mid lateral wall	TTE	Basal, lateral wall (severe hypokinesis) Apical wall (Hyper-reflective/ Increased echogenicity)
4	CM -- Hypertrophic cardiomyopathy and scar assessment	AICD	Basal, Mid inferior, Inferolateral, Anterolateral LV wall	TTE	Asymmetric septal hypertrophy.
5	CAD -- Scar mapping for ventricular tachycardia ablation	AICD	Basal, Mid inferolateral wall	TTE	Basal Mid inferolateral wall (Hyper-reflective/ increased echogenicity)
6	CM -- Idiopathic cardiomyopathy and Worsening ventricular arrhythmias.	AICD	Mid to apical anterior, Anteroseptal, Anterolateral LV wall	TTE	Apical (Akinesis) Anteroseptal (Akineis) Anterolateral wall (Hypokinesis) Septum and apex (Hyper-reflectivity/Increased echogenicity)
7	CM -- HCM, recurrent AICD shocks	AICD	Mid myocardium in anteroseptal Basal wall	TTE	Marked asymmetric LV hypertrophy.
8	CM -- Progression of Sarcoidosis	Permanent	Subepicardial portion of antero-septal mid ventricular wall Mid myocardial basal, Anteroseptal wall and Sub endocardial mid inferolateral wall.	TTE	Mid myocardial wall (Hypokinesis) Inferoposterior (Hyper-reflective/increased echogenicity)
		Pacemaker			
9	CAD -- Ventricular tachycardia storm; scar assessment	AICD	Inferior wall and Basal to apical LV, Anterior wall at basal, mid ventricular level.	TTE	Inferior, septal, apical (Akinesis) Inferior and posterior wall. (Hyper-reflective/increased echogenicity)

*AICD* = Automated Implantable Cardioverter Defibrillator, *CAD* = Coronary artery disease, *CM* = Cardiomyopathy, *C-MRI* = cardiac *MRI, DE* = Delayed enhancement, *HCM* = Hypertrophic cardiomyopathy, *LV* = Left ventricle, *PM* = Pace maker, *TTE* = Transthoracic echocardiogram, *VT* = Ventricular Tachycardia.

MDE images were acquired at a mean of 6.6 +/- 3.1 minutes after a mean contrast dose of 147 +/-15 ml. Decreased tube voltage was used in all patients (100 kVp, n=11, 80 kVp, n=7). The imaging parameters of the MDE scans are summarized in Table 3. All CT-MDE images were deemed interpretable and of diagnostic quality by the clinical reader.

**Table 3:** Imaging parameters

**Tube voltage**	**100 kVp: 11 patients, 80 kVp: 7 patients**
Tube Current (mAs)	669 +/- 229
Contrast amount (ml)	147 +/-15
Timing of Scan (minutes after injection)	6.6 +/- 3.1
Contrast to Noise ratio (CNR)	1.6 +/- 0.7
Effective radiation dose (mSv)	4.1+/- 2.6

At the time of cardiac CT, patients had either a contradiction to MRI (n=15), or a prior suboptimal or unsuccessful cardiac MRI (n=3) (Table 4). Nine of the 18 cases were reported as positive for CT-MDE. Out of these nine positive cases, CT scans were performed for assessment of ischemic myocardial scar in five patients. Of the remaining four positive cases, two were performed to assess suspected infiltrative disease; one was performed for scar identification in hypertrophic cardiomyopathy, and one for scar identification in non-ischemic dilated cardiomyopathy.

**Table 4:** Shows patient distribution and indications for the delayed enhancement with Cardiac CT (AICD = Implantable cardiac defibrillator, MDE = Myocardial Delayed Enhancement)

		
AICD / Pacemaker	8	5
Non diagnostic/ inconclusive cardiac MRI	1	2
Patient discomfort/Claustrophobia	0	1
Contrast contraindication (Gadolinium)	0	1

All of the nine positive cases had echocardiograms available for comparison, each of which was performed within 12 weeks of the CT exam. Two patients had previous cardiac MRIs (within 1 week and 12 weeks of the CT) and one patient had additional SPECT-MPI (within 1 week of the CT) for comparison.

In one patient, the wall motion assessment by cardiac CT was not possible due to the use prospectively ECG-triggered technique.

A total of 136 segments were analyzed for wall motion abnormality; 32 of these segments were clinically interpreted as positive for CT-MDE. The kappa statistics for the agreement of wall motion abnormality between 2-dimensional echocardiography and wall motion abnormality by cardiac CT for all segments was 0.83 and the kappa statistics for agreement of segments with abnormal wall motion between 2-dimensional echocardiography and segments with abnormal CT-MDE with cardiac CT was 0.82 (Tables 5 and 6).

**Table 5:** Relative agreement of all segments between 64-slice MDCT and echocardiography scores

**All segment**		**Echocardiography score**			
**n=136**		**0**	**1**	**2**	**Total**
CT score	0	64	4	0	68
	1	3	42	1	46
	2	4	2	16	22
Total		71	48	17	136

Kappa = 0.83.

Wall motion by MDCT and echocardiography showed excellent agreement (k=0.83).

Wall motion scores of 0 to 2 were assigned to the different segments. 0 = normal wall motion; 1 = hypokinesia; 2 = Akinesia, MDCT, multidetector CT.

**Table 6:** Relative agreement of CT DE positive only segments between 64-slice MDCT and echocardiography scores

**D+segment**		**Echocardiography score**			
**n=136**		**0**	**1**	**2**	**Total**
CT score	0	15	2	0	17
	1	1	13	0	14
	2	0	0	1	1
Total		16	15	1	32

Kappa = 0.82.

Wall motion by MDCT and echocardiography showed excellent agreement (k=0.82).

Wall motion scores of 0 to 2 were assigned to the different segments. 0 = normal wall motion; 1 = hypokinesia; 2 = Akinesia. DE, delayed enhancement, MDCT, multidetector CT.

**Figure 1:**
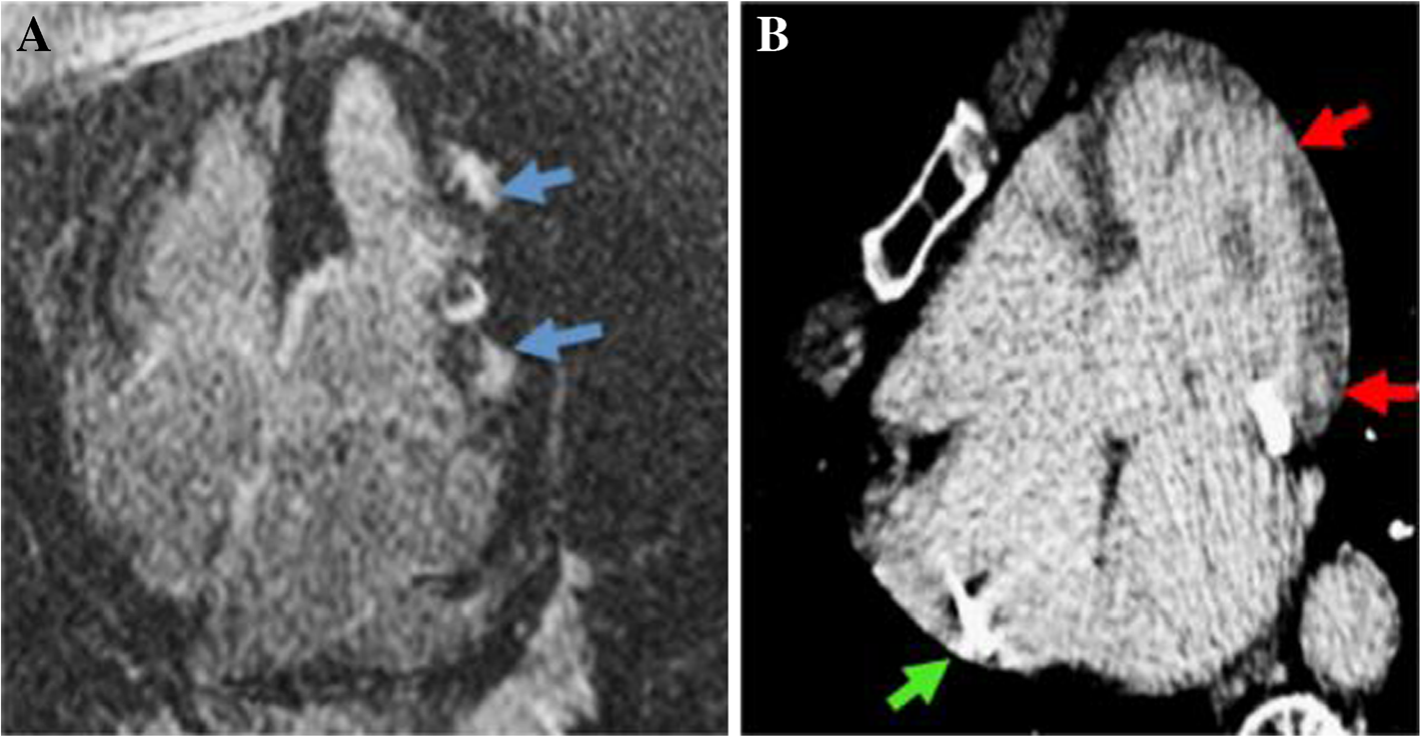
**Panel A. 72 year old female with known sarcoidosis was found to have patchy areas of delayed hyper enhancement (blue arrows) in the lateral wall of the left ventricle at cardiac MRI.** Panel **B**. Three months after pacemaker placement which precluded repeat MRI (green arrow), the patient developed symptoms warranting analysis for cardiac sarcoid progression. MDE by cardiac CT shows corresponding patches of delayed hyperenhancement (red arrows) which confirmed stable involvement since the recent cardiac MRI.

**Figure 2:**
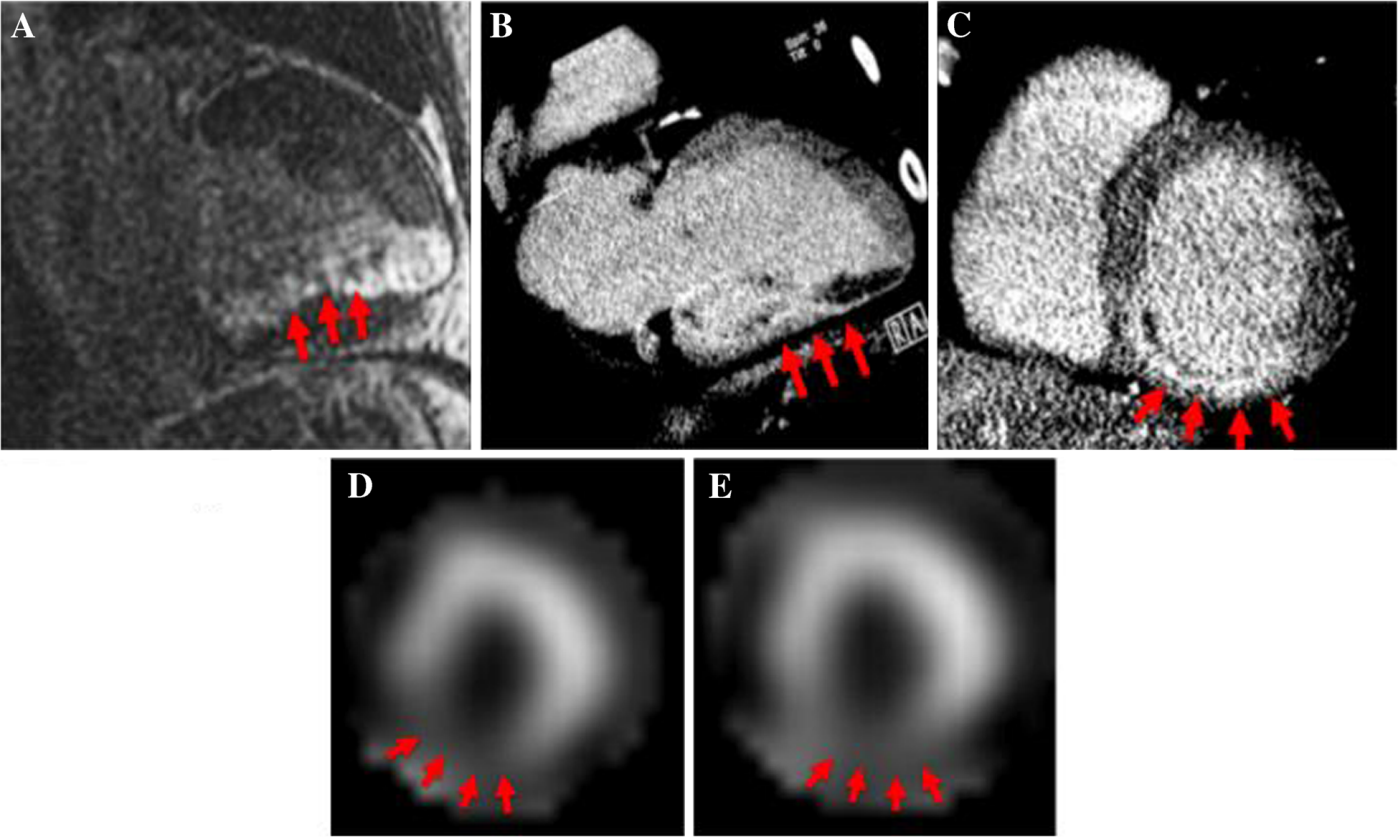
**A 56-year-old male with hyperlipidemia presented with atypical chest pain.** Cardiac MRI demonstrated scar (abnormal delayed enhancement--red arrows) in the entire inferior wall of the left ventricle as seen in two-chamber view (Panel **A**). CTA was performed to assess for previously unsuspected coronary disease; delayed images were performed confirming the same inferior wall delayed enhancement as seen in the two chamber and short-axis views (Panels **B** &**C**). The same patient underwent stress and rest Technitium-99m-Sestamibi SPECT- MPI (short axis images obtained at stress-Panel **D** and rest-Panel **E**), which corroborated the fixed defect in the inferior wall.

In the two cases positive for CT-MDE in which a prior cardiac MRI was available, the kappa statistics for agreement between the segments showing MDE with CT and MRI was 1.0 (i.e. in both cases, the same segments were positive for delayed enhancement on both exams) (Figures 1 and 2).

In the single case for which SPECT-MPI was available, there was a fixed perfusion defect in the same segments in which the cardiac CT identified CT-MDE. The kappa statistics for agreement between the segments of CT-MDE and SPECT-MPI was therefore 1.0 (Figure 2).

In the cases reported as negative for CT-MDE (n=9), each had concordant imaging and no abnormal segments were found by other imaging modalities.

## Discussion

Cardiac MRI is the standard of reference for scar detection and myocardial characterization with late gadolinium enhancement, also known as myocardial delayed enhancement imaging (MR-MDE). However, numerous situations arise in routine practice whereby cardiac MRI is contraindicated, or not feasible. When the detection of myocardial scar / injury is clinically relevant in these patients, a cardiac CT-MDE protocol may offer a practical alternative. Our data demonstrates that while delayed imaging for myocardial indications with cardiac CT in clinical practice is requested infrequently, the presence of abnormal delayed enhancement in myocardial segments with cardiac CT has an excellent correlation with regional abnormalities detected on other concurrent cardiac imaging exams.

To our knowledge, this is the largest series of clinical cases imaged with CT-MDE for both ischemic and non-ischemic causes. Although pathologic proof of scar or other corresponding myocardial abnormalities was not obtained, comparison of the abnormal segments by other modalities (echocardiography, SPECT-MPI, and cardiac MRI) demonstrated that in all cases, the myocardial segments with positive MDE were abnormal by at least one other imaging modality. This suggests that the CT evidence of MDE was due to a true myocardial abnormality. Several animal and human studies have demonstrated that delayed CT imaging can identify myocardial scar [8-10].

All patients referred for cardiac CT had a contraindication to MRI or an unsuccessful cardiac MR exam. All modern CT scanners are capable of imaging the heart and are widely available. However, several challenges exist. MRI-MDE benefits from high contrast-to-noise ratios, made possible by “nulling” of signal in the normal, healthy myocardium at delayed image acquisition. The inability of CT to null normal myocardium results in significantly lower CNR values on MDE images with CT [5]. While the CNR values calculated in our study are indeed low, the image quality was deemed adequate and clinically interpretable in all cases. Also, the use of radiation necessary for cardiac CT is a concern. However, newer CT systems and consistent use of existing dose reduction strategies can lower the radiation dose per acquisition to sub-mSv levels [11,12].

The chief limitation of our study is the retrospective design. Any analysis of clinical cases performed for various indications during a 5-year period will result in heterogeneous scan techniques and equipment. Tube voltage, tube current, and radiation doses were selected according to the available scanner, patient body habitus, heart rate, and the clinical indication. Despite these variations, we found that the studies all had interpretable image quality.

Another limitation of our study is a small number of clinical patients in whom CT-MDE protocol was performed. These scans were performed only as a last resort to answer a difficult clinical question. Although the results are encouraging, it is important to note that each case was an off-label use of contrast and a non-routine, unproven utilization of cardiac CT.

If confirmed in large-scale studies, myocardial characterization with delayed enhancement may eventually become an accepted indication for cardiac CT. Validation of this application of cardiac CT could perhaps lead to the development of comprehensive cardiac CT protocols demonstrating coronary artery anatomy, ventricular function, and scar detection. This rich dataset could be obtained in a single brief examination, facilitating diagnoses and guiding patient management [13,14]. Abnormal delayed enhancement has been shown to provide incremental diagnostic value to other cardiac MRI findings, and cardiac CT with MDE imaging could bring this diagnostic benefit to an even greater number of patients [15].

## Abbreviations

CT: Computed Tomography; MDE: Myocardial Delayed Enhancement; MPI: Myocardial Perfusion Imaging; MRI: Magnetic Resonance Imaging; SPECT: Single Photon Emission Computed Tomography; TTE: Transthoracic Echocardiogram.

## Competing interests

The authors declare that they have no competing interests.

## Authors’ contributions

BBG and MSS = equal contributions in manuscript preparation and revision. SU = CT image review, NK = ECHO image review, LCE = collection of the images, HMZ & VV = Formulation of tables and data analysis, YV = Concept analysis, UH & RCC = critique of the manuscript, SA = manuscript editing, TJB = manuscript, editing and approval for submission. All authors read and approved the final manuscript.
